# Locally injected ivabradine inhibits carrageenan-induced pain and inflammatory responses via hyperpolarization-activated cyclic nucleotide-gated (HCN) channels

**DOI:** 10.1371/journal.pone.0217209

**Published:** 2019-05-24

**Authors:** Saki Miyake, Hitoshi Higuchi, Yuka Honda-Wakasugi, Maki Fujimoto, Hotaka Kawai, Hitoshi Nagatsuka, Shigeru Maeda, Takuya Miyawaki

**Affiliations:** 1 Department of Dental Anesthesiology and Special Care Dentistry, Okayama University Graduate School of Medicine, Dentistry and Pharmaceutical Sciences, Okayama, Japan; 2 Department of Dental Anesthesiology, Okayama University Hospital, Okayama, Japan; 3 Department of Oral Pathology and Medicine, Okayama University Graduate School of Medicine, Dentistry and Pharmaceutical Sciences, Okayama, Japan; Boston Children’s Hospital and Harvard Medical School, UNITED STATES

## Abstract

**Background:**

Recently, attention has been focused on the role of hyperpolarization-activated cyclic nucleotide-gated (HCN) channels in the mechanism of and as a treatment target for neuropathic and inflammatory pain. Ivabradine, a blocker of HCN channels, was demonstrated to have an effect on neuropathic pain in an animal model. Therefore, in the present study, we evaluated the effect of ivabradine on inflammatory pain, and under the hypothesis that ivabradine can directly influence inflammatory responses, we investigated its effect in *in vivo* and *in vitro* studies.

**Methods:**

After approval from our institution, we studied male Sprague–Dawley rats aged 8 weeks. Peripheral inflammation was induced by the subcutaneous injection of carrageenan into the hindpaw of rats. The paw-withdrawal threshold (pain threshold) was evaluated by applying mechanical stimulation to the injected site with von Frey filaments. Ivabradine was subcutaneously injected, combined with carrageenan, and its effect on the pain threshold was evaluated. In addition, we evaluated the effects of ivabradine on the accumulation of leukocytes and TNF-alpha expression in the injected area of rats. Furthermore, we investigated the effects of ivabradine on LPS-stimulated production of TNF-alpha in incubated mouse macrophage-like cells.

**Results:**

The addition of ivabradine to carrageenan increased the pain threshold lowered by carrageenan injection. Both lamotrigine and forskolin, activators of HCN channels, significantly reversed the inhibitory effect of ivabradine on the pain threshold. Ivabradine inhibited the carrageenan-induced accumulation of leukocytes and TNF-alpha expression in the injected area. Furthermore, ivabradine significantly inhibited LPS-stimulated production of TNF-alpha in the incubated cells.

**Conclusion:**

The results of the present study demonstrated that locally injected ivabradine is effective against carrageenan-induced inflammatory pain via HCN channels. Its effect was considered to involve not only an action on peripheral nerves but also an anti-inflammatory effect.

## Introduction

Neuropathic pain is a chronic pain state, and it frequently impairs patients’ quality of life [1–4]. Many investigations have been conducted on its mechanism and treatment, but the mechanism is complex and remains to be fully clarified [5–8]. Furthermore, not only direct nerve injury but also other conditions, such as inflammation and viral infection, can cause neuropathic pain and increase the complexity [6–8]. Various kinds of drugs, including antiepileptic drugs, antidepressants, pregabalin, N-methyl-D-aspartate (NMDA) receptors blockers, NSAIDs, and opioids are currently used as treatments targeting neuropathic pain, but these drugs may not be sufficient for relief from neuropathic pain [9, 10].

Recently, attention has been focused on the role of hyperpolarization-activated cyclic nucleotide-gated (HCN) channels in the mechanisms of neuropathic pain and as a treatment target [11, 12]. HCN channels are distributed in various tissues, being expressed in cardiac tissue, brain tissue, and peripheral neurons [13–16]. Their activation following hyperpolarization of the cellular membrane contributes to their role in setting the membrane potential and generating spontaneous activity in excitable cells [17, 18]. Recent evidence suggested that the current passing through HCN channels contributes to abnormal peripheral nerve activity after axonal injury [19]. HCN channels consist of four isoforms (HCN1-4). All four HCN isoforms are expressed in the central nervous system (CNS) and peripheral nerves [20].

There are specific blockers of HCN channels, including ZD7288 and ivabradine [21]. Recent evidence demonstrated that ZD7288 and ivabradine act on peripheral sensory neurons and have inhibitory effects on neuropathic pain in an animal model [19, 22]. Ivabradine is clinically used as an anti-anginal and cardiotonic agent, acting via HCN4 channels in the heart [21, 23]. Therefore, ivabradine could be expected as a new medicine for neuropathic pain, which has a different mechanism of pain control than current therapies. Ivabradine and ZD7288 were also demonstrated to have an inhibitory effect on inflammatory and neuropathic pain [11, 12, 24, 25].

Acute inflammation is characterized by the accumulation of leukocytes and macrophages and accelerated by the release of inflammatory mediators, including cytokines, PGE2, serotonin, and bradykinin [26, 27]. The increase of these mediators leads to the development of neuropathic pain [22]. The occurrence of neuropathic pain caused by inflammation can delay the recovery of patients and may lead to chronic pain in some patients. Thus, the treatment of neuropathic pain is clinically significant in patients with acute inflammation. HCN channels are involved in the modulation of inflammatory pain [20]. Therefore, the primary purpose of the present study was to evaluate the effect of ivabradine on inflammatory pain. ZD7288 was demonstrated to have an effect on neuropathic pain following local injection [22]. Therefore, we investigated the effect of locally injected ivabradine in the animal model we previously used. Furthermore, we hypothesized that HCN channel blockers directly influence inflammatory responses, so we evaluated the effect of ivabradine on inflammatory responses *in vivo*, and further investigated its direct effect on the production of an inflammatory mediator, TNF-alpha, in mouse macrophage-like cells *in vitro*.

## Materials and methods

### Animals

The protocol of the present study was approved by the Animal Care and Use Committee of Okayama University (Approval No. OKU-2017025, OKU-2018059, OKU-2018202). We studied male Sprague–Dawley rats, aged 8 weeks (weight: 250–340 g), which were obtained from Charles River Laboratories (Osaka, Japan). Rats were housed in steel cages in a room kept at 24°C with 50 ± 10% relative humidity under a 12-hour cycle of light and dark and fed a laboratory diet (CE-II, CLEA, Tokyo, Japan). Water was freely available. This study was conducted in accordance with the Guidelines for Animal Experiments at Okayama University Advanced Science Research Center.

### Agents

Lambda-carrageenan (carrageenan) was purchased from Santa Cruz Biotechnology, Inc. (Dallas, TX, USA) and used as a 1% (weight/volume) solution [28]. Ivabradine hydrochloride was purchased from Tokyo Chemical Industry Co. (Tokyo, Japan). ZD7288, lamotrigine, forskolin, and lipopolysaccharide (LPS, from *Escherichia coli* O55:B5) were purchased from Sigma-Aldrich (St. Louis, MO, USA). ZD7288 is a pyridinium derivative, widely used as pharmacological tool to study HCN channels. Lamotrigine and forskolin activate HCN channels [24, 29]. Carrageenan, ivabradine, and ZD7288 were diluted with physiological saline. Stock solutions of lamotrigine and forskolin were prepared in dimethylsulfoxide (DMSO) and dissolved before use in external media to a final concentration containing no more than 0.1% DMSO.

### Animal model of peripheral inflammation

Peripheral inflammation was induced by an injection of carrageenan at a volume of 50 μL into the right hindpaw of rats with a 27-gauge needle under inhalation anesthesia with isoflurane. The degree of nociception after injection of the test solutions was evaluated by measuring the paw withdrawal response on applying mechanical stimulation with von Frey filaments (TACTILE TEST (AESTHESIO), Semmes-Weinstein Von Frey Anesthesiometer, Muromachi Kikai CO., Tokyo, Japan). Rats were placed on a metal mesh floor in individual clear plastic cages. After adaptation to the environment, the plantar surface of the hindpaw was touched vertically with a series of von Frey filaments (0.4, 0.6, 1, 1.4, 2, 4, 6, 8, and 15 g). Each trial was started with 2 g for 2–3 s. A brisk withdrawal or flinching of the paw was considered a positive response. The 50% withdrawal threshold (pain threshold) was determined using the up-and-down method [30]. In the absence of a positive response to a filament, a stronger stimulus was applied, whereas, in the presence of a positive response, the next weaker stimulus was applied. The resulting pattern of positive and negative responses was tabulated using the convention: X = positive; O = negative, and the 50% response threshold was interpolated using the following formula:

$$50\%\;\text{g}\;\text{threshold}=\frac{10^{\text{Xf}+\kappa \delta }}{10,000}$$

where Xf = value (log units) of the final von Frey filament used, κ = tabular value for the pattern of positive/negative responses [30], and δ = mean difference (log units) between stimuli (here, 0.224).

The investigator who injected the test solutions was blinded to the solutions being administered, and the investigator who measured the withdrawal threshold was also blinded to them.

### Evaluation of pain threshold

Ivabradine was subcutaneously injected at a final concentration of 10, 20, or 50 μM into the right hindpaw of rats, combined with 1% carrageenan. The pain threshold was evaluated 2, 4, 6, and 8 hours after the injection. Furthermore, to ensure that the action of ivabradine was mediated via HCN channels, we subcutaneously injected lamotrigine or forskolin, an activator of HCN channels, at a final concentration of 10 μM, combined with 1% carrageenan or 1% carrageenan plus ivabradine at 20 μM, and the effect of lamotrigine or forskolin on ivabradine's action was evaluated. As a control, only 1% carrageenan was subcutaneously injected into the hindpaw of rats.

### Histological evaluation

For histological evaluation, physiological saline, 1% carrageenan, and 1% carrageenan plus ivabradine at a concentration of 20 and 50 μM were injected into the paws of rats, respectively. The rats were euthanized by the excess administration of isoflurane 2 and 4 hours after the injection, and the injected area of the paw was excised as a sample. The sample was fixed in 10% neutral buffered formalin and embedded in paraffin, and sections were cut at a thickness of 5 μm for hematoxylin-eosin staining. The accumulation of leukocytes in the injected area was observed as a histological finding using a microscope. Furthermore, we histologically evaluated the number of leukocytes using the same methods as described in a previous study [31]. The evaluation was performed with a light binocular microscope and included a description of the observed tissue response. Additionally, 5 fields around each osseous defect were randomly captured using the x 200 magnification of a binocular microscope fixed with a charged coupled device camera to evaluate the number of leukocytes in the histologic sections. The number of leukocytes was counted on each field image. Leukocytes were differentiated from the other cells based on the morphologic characteristics of nuclei. We defined a foliaceous nucleus as a neutrophil and a rotund nucleus as a lymphocyte, and counted them as leukocytes. We evaluated the effects of ivabradine on the accumulation of leukocytes, compared with that of only carrageenan-injected samples. Furthermore, we subcutaneously injected forskolin, at a final concentration of 10 μM, combined with 1% carrageenan plus ivabradine at 20 μM, and the effect of forskolin on ivabradine's action was evaluated, compared with that of 1% carrageenan plus ivabradine at 20 μM.

### Immunohistochemistry of TNF-alpha expression in rats

The evaluation of TNF-alpha expression was performed using the same methods as described in a previous study [31]. We observed paw tissue samples in the injected area 2 hours after the injection of physiological saline, only 1% carrageenan, 1% carrageenan plus ivabradine at concentrations of 20 and 50 μM, and 1% carrageenan plus ivabradine at 20 μM plus forskolin at 10 μM. Sections (3 μm) were collected from the obtained samples and mounted on salinized slides for immunohistochemistry. In brief, sections were deparaffinized in a series of xylene for 15 minutes and rehydrated in graded ethanol solution. Endogenous peroxidase activity was blocked by incubating the sections in 0.3% H2O2 in methanol for 30 minutes. Antigen retrieval was achieved by heat treatment using 10-mM citrate buffer solution at pH 6.0. After treatment with normal serum, the sections were incubated with the primary antibodies for TNF-alpha (LifeSpan BioSciences, Seattle, WA, USA) at 4°C. Tagging of the primary antibody was achieved using the Vectastain ABC kit (Vector Labs, Burlingame, CA, USA). Visualization of immunoreactivity was performed by developing the enzyme complex with diaminobenzidine/H2O2 solution (Histofine DAB substrate, Nichirei, Tokyo, Japan) and counterstained with Mayer hematoxylin.

### Cell culture

RAW264.7 mouse macrophage-like cells (DS Pharma Biomedical, Osaka, Japan) were cultured in D-MEM (DS Pharma Biomedical, Osaka, Japan) supplemented with 10% fetal bovine serum (FBS) and 2 mM L-glutamine in 100-mm dishes. All cells were maintained at 37°C and 5% CO2 in a humidified atmosphere. The cells were harvested with 0.25% trypsin–EDTA, added to a 12-well plate at a density of 1×10^6^ cells/well, and cultured overnight at 37°C in the fresh medium.

### Evaluation of TNF-alpha production in the cells

The cells were incubated with LPS at 10 ng/mL and ivabradine at concentrations of 10, 20, and 50 μM or ZD7288 at concentrations of 10 and 50 μM and at 37°C for 2, 4, and 6 hours in a 12-well plate (1 mL/well). After the incubation, the supernatants of the cells were collected, and the TNF-alpha concentration was measured using a specific ELISA kit (Thermo Fisher Scientific, Waltham, MA, USA). Furthermore, we evaluated whether forskolin at a concentration of 10 μM reversed the effect of ivabradine at 20 μM on TNF-alpha production.

### Statistical analysis

We used one- or two-way analysis of variance (ANOVA) followed by post-hoc Tukey’s multiple comparisons test, Sidak’s multiple comparisons test, or Dunnett’s multiple comparisons test. All statistical analyses were performed using statistical analysis software (GraphPad Prism ver. 4R). P< 0.05 was regarded as significant. The data are presented as the mean ± standard deviation.

## Results

### The effect of ivabradine on the pain threshold

**Fig 1:**
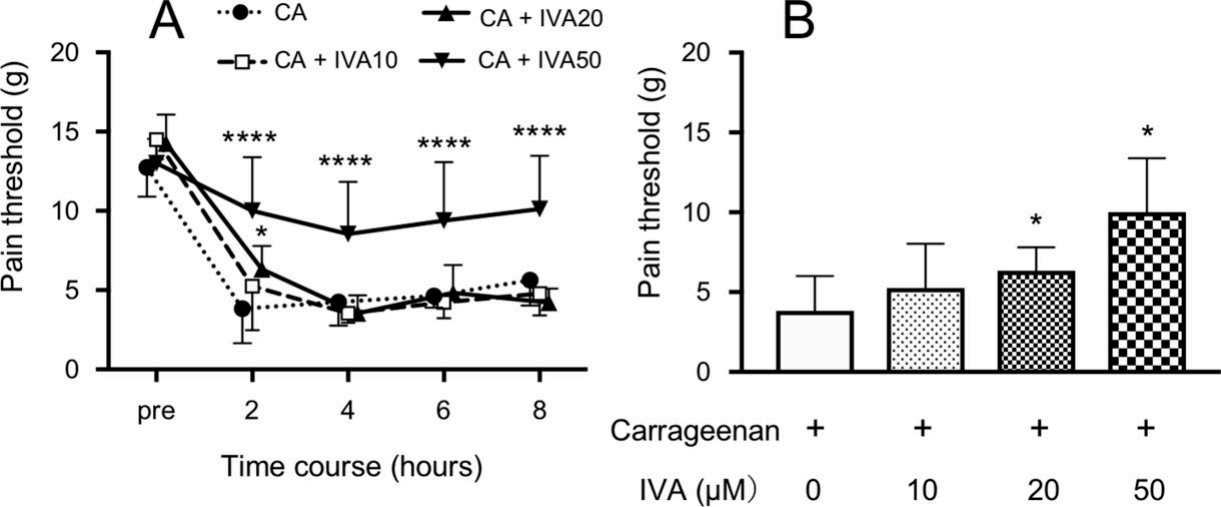
The effect of ivabradine (IVA) on the carrageenan-induced 50% paw withdrawal threshold (pain threshold) in rats. Carrageenan at 1% was subcutaneously injected into the hindpaw, causing acute inflammation. IVA at different concentrations was subcutaneously injected into the hindpaw, combined with carrageenan. (A) The time-course of the pain threshold until 8 hours after the injection of test solutions (CA: only carrageenan; CA + IVA10: carrageenan + IVA at 10 μM; CA + IVA20: carrageenan + IVA at 20 μM; CA + IVA50: carrageenan + IVA at 50 μM). (B) The pain threshold 2 hours after the injection of test solutions. *P< 0.05, ****P< 0.0001 compared with CA. Data represent the mean ± SD (n = 6 for each).

Before the injection of carrageenan, the pain threshold was a cut-off value of 15 g in almost all rats. The time-courses of the pain threshold until 8 hours after the injection of test solutions are shown in Fig 1A. After the injection of carrageenan, the pain threshold decreased until 8 hours. The values 2 hours after the injection of carrageenan plus ivabradine at 20 and 50 μM were significantly higher than those of only carrageenan, and the values 4, 6, and 8 hours after the injection of carrageenan plus ivabradine at 50 μM were significantly higher than those of only carrageenan. Furthermore, ivabradine dose-dependently increased the pain threshold 2 hours after the injection, and the values of ivabradine at 20 and 50 μM were significantly higher than that of only carrageenan (Fig 1B).

### The effects of activators of HCN channels on the action of ivabradine

**Fig 2:**
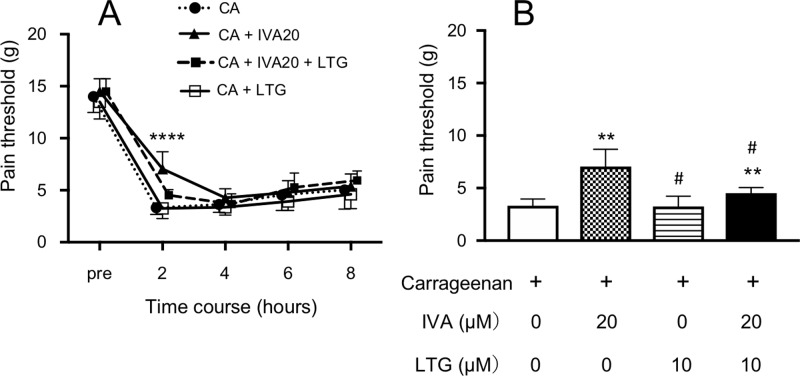
The effect of lamotrigine (LTG) to reverse the action of ivabradine (IVA) on the carrageenan-induced 50% paw withdrawal threshold (pain threshold) in rats. Carrageenan at 1% was subcutaneously injected into the hindpaw, causing acute inflammation. IVA at 20 μM and/or LTG at 10 μM was injected into the hindpaw combined with carrageenan. (A) The time-course of the pain threshold until 8 hours after the injection of test solutions (CA: only carrageenan; CA + IVA20: carrageenan + IVA at 20 μM; CA + IVA20 + LTG: carrageenan + IVA at 20 μM + LTG at 10 μM; CA + LTG: carrageenan + LTG at 10 μM). (B) The pain threshold 2 hours after the injection of test solutions. **P< 0.01, ****P< 0.0001 compared with CA. # P< 0.05 compared with CA + IVA20. Data represent the mean ± SD (n = 6 for each).

Fig 2A and 2B show the time-course and 2-hour effects of lamotrigine at 10 μM on the action of ivabradine at 20 μM on the pain threshold, respectively. The value 2 hours after the injection of carrageenan plus ivabradine was significantly higher than that of only carrageenan, but it was significantly decreased by the addition of lamotrigine (Fig 2B). Thus, lamotrigine significantly reversed the inhibitory effect of ivabradine on the pain threshold.

**Fig 3:**
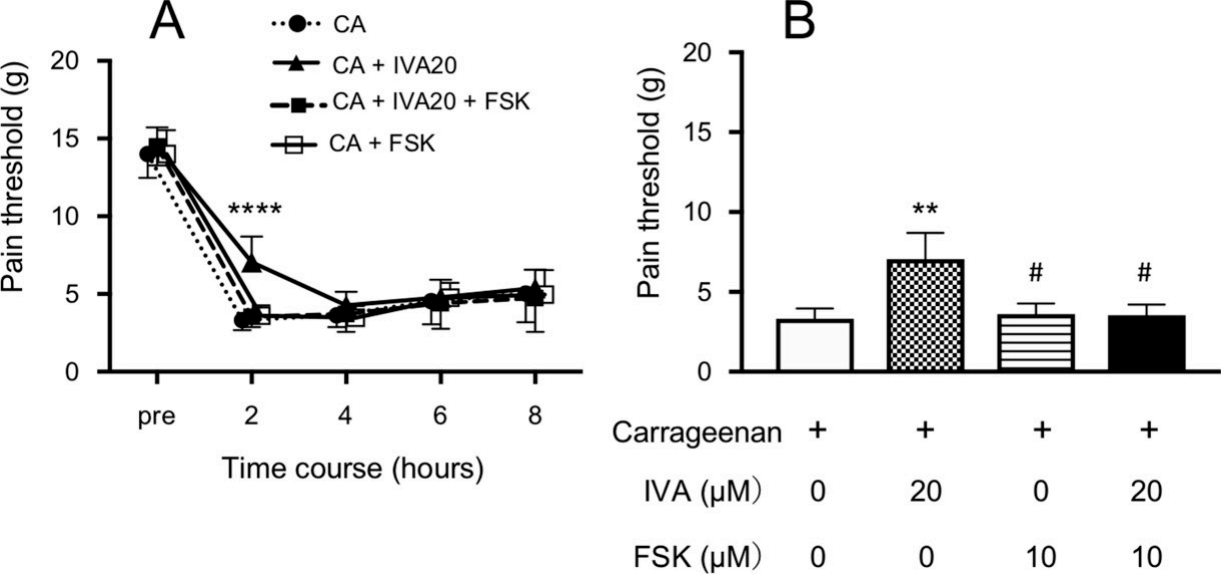
The effect of forskolin (FSK) to reverse the action of ivabradine (IVA) on the carrageenan-induced 50% paw withdrawal threshold (pain threshold) in rats. Carrageenan at 1% was subcutaneously injected into the hindpaw, causing acute inflammation. IVA at 20 μM and/or FSK at 10 μM was injected into the hindpaw combined with carrageenan. (A) The time-course of the pain threshold until 8 hours after the injection of test solutions (CA: only carrageenan; CA + IVA20: carrageenan + IVA at 20 μM; CA + IVA20 + FSK: carrageenan + IVA at 20 μM + FSK at 10 μM; CA + FSK: carrageenan + FSK at 10 μM). (B) The pain threshold 2 hours after the injection of test solutions. **P< 0.01, ****P< 0.0001 compared with CA # P< 0.05 compared with CA+ IVA20. Data represent the mean ± SD (n = 6 for each).

Similarly, Fig 3A and 3B show the time-course and 2-hour effects of forskolin at 10 μM on the action of ivabradine at 20 μM on the pain threshold, respectively. The pain threshold value of carrageenan plus ivabradine was significantly higher than that of only carrageenan 2 hours after the injection, but there were no differences in pain threshold values with only carrageenan, carrageenan plus forskolin, and carrageenan plus ivabradine plus forskolin (Fig 3B). Thus, forskolin significantly reversed the inhibitory effect of ivabradine on the pain threshold.

### The effect of a specific HCN channel blocker, ZD7288, on the pain threshold

**Fig 4:**
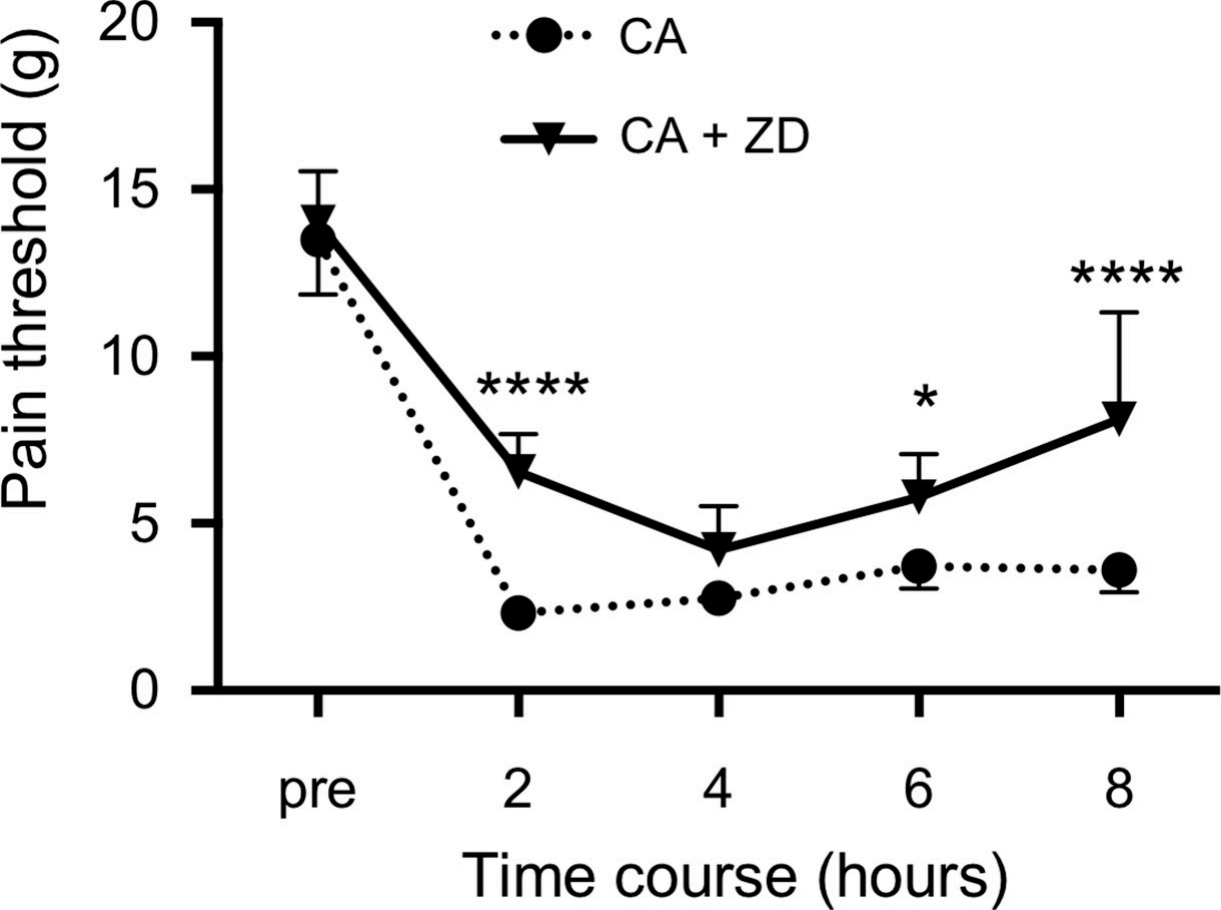
The effect of ZD7288 on the carrageenan-induced 50% paw withdrawal threshold (pain threshold) in rats. Carrageenan at 1% was subcutaneously injected into the hindpaw, causing acute inflammation. ZD7288 at 50 μM was subcutaneously injected into the hindpaw combined with carrageenan. The figure shows the time-course of the pain threshold until 8 hours after the injection of test solutions (CA: only carrageenan; CA + ZD: carrageenan + ZD7288 at 50 μM). *P< 0.05, ****P< 0.0001 compared with only carrageenan. Data represent the mean ± SD (n = 6 for each).

The time-course of the pain threshold until 8 hours after the injection of ZD7288 at 50 μM is shown in Fig 4. ZD7288 increased the pain threshold 2, 6, and 8 hours after the injection as well as ivabradine, compared with only carrageenan injection. The values 2, 6, and 8 hours after the injection of carrageenan plus ZD7288 were significantly higher than those of only carrageenan (Fig 4).

### The effect of ivabradine on inflammatory responses in rats

**Fig 5:**
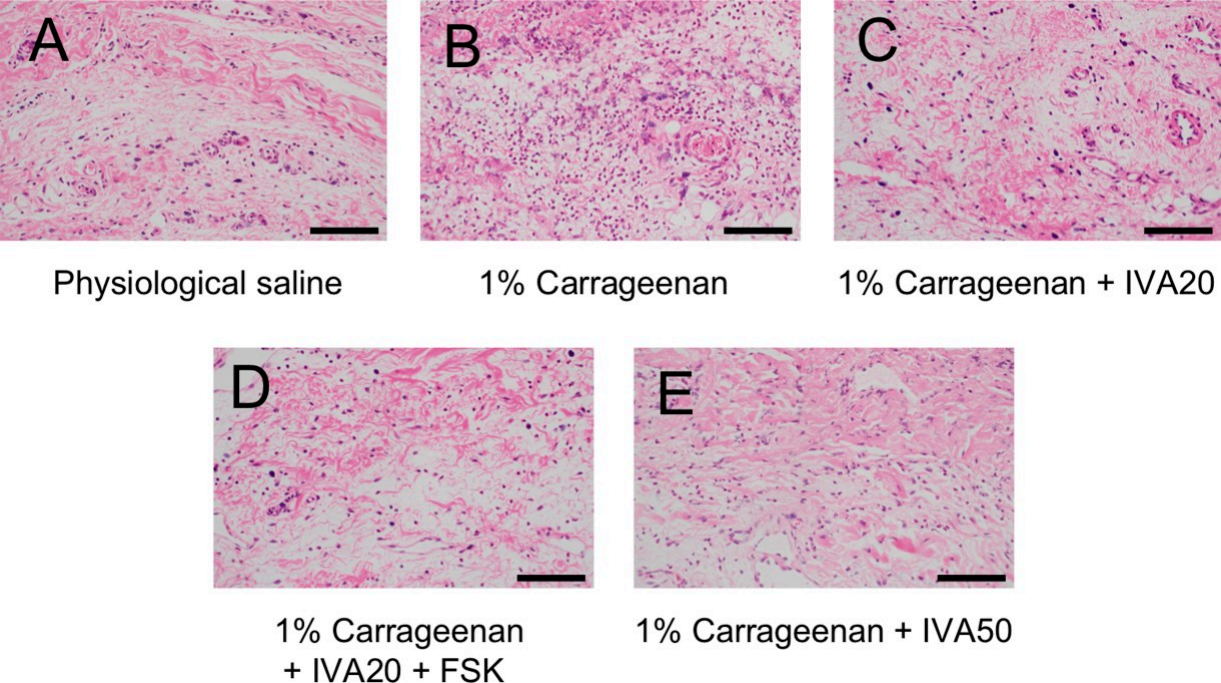
Hematoxylin-eosin staining findings in the hindpaw area of rats (2 hours after injection). The following test solutions were subcutaneously injected into the hindpaw: physiological saline (A), 1% carrageenan (B), 1% carrageenan + ivabradine (IVA) at 20 μM (C), 1% carrageenan + IVA at 20 μM + forskolin at 10 μM (FSK) (D), and 1% carrageenan + IVA at 50 μM (E). Scale bar represents 200 μm.

**Fig 6:**
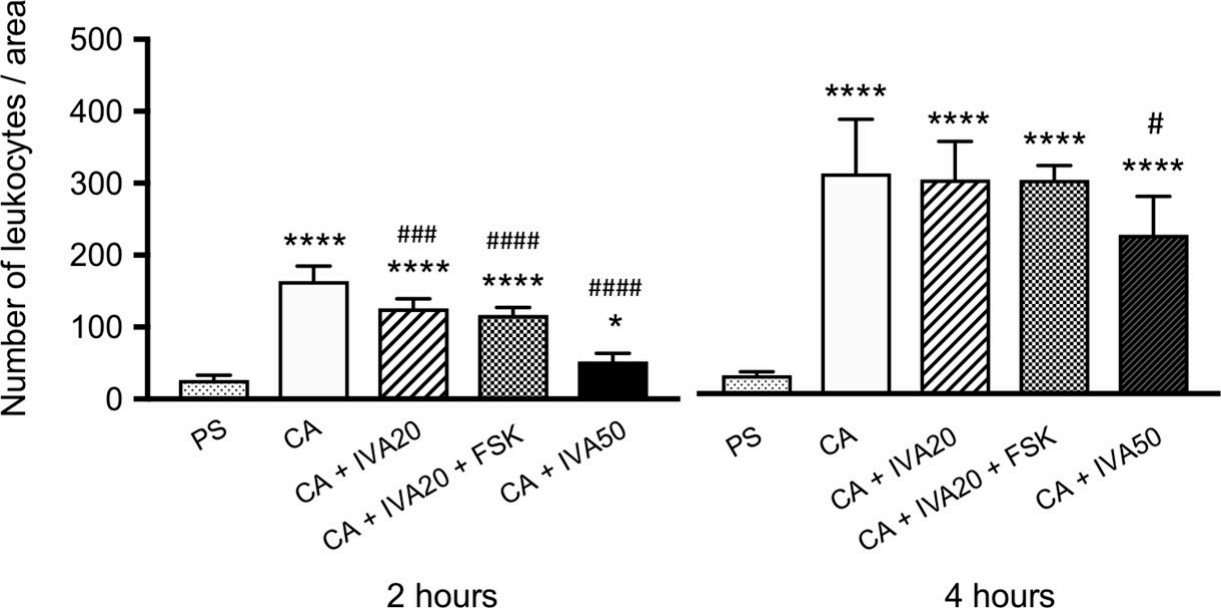
The effect of ivabradine (IVA) on carrageenan-induced accumulation of leukocytes in the hindpaw of rats. The following test solutions were subcutaneously injected into the hindpaw: physiological saline (PS), 1% carrageenan (CA), CA + IVA at 20 μM (IVA20), CA + IVA20 + forskolin at 10 μM (FSK), and CA + IVA at 50 μM (IVA50). *P< 0.05, ****P< 0.0001 compared with physiological saline. # P< 0.05, ### P< 0.001, #### P< 0.0001 compared with CA. Data represent the mean ± SD (n = 6 for each).

In the samples collected 2 and 4 hours after the injection of carrageenan, a greater accumulation of leukocytes was histologically observed than that after the physiological saline injection, while the addition of ivabradine at 20 and 50 μM to carrageenan inhibited the accumulation of leukocytes in the injected area (Fig 5). Quantitatively analyzing the accumulation of leukocytes, carrageenan increased the number of leukocytes in the injected area, and ivabradine at 20 μM significantly lowered it at 2 hours after the injection, while ivabradine at 50 μM significantly lowered it at both 2 and 4 hours after the injection (Fig 6). Thus, ivabradine at 20 and 50 μM significantly inhibited the carrageenan-induced accumulation of leukocytes at 2 and 4 hours after the injection. However, forskolin at 10 μM did not reverse the inhibitory effect of ivabradine at 20 μM on the inflammatory response (Figs 5 and 6).

**Fig 7:**
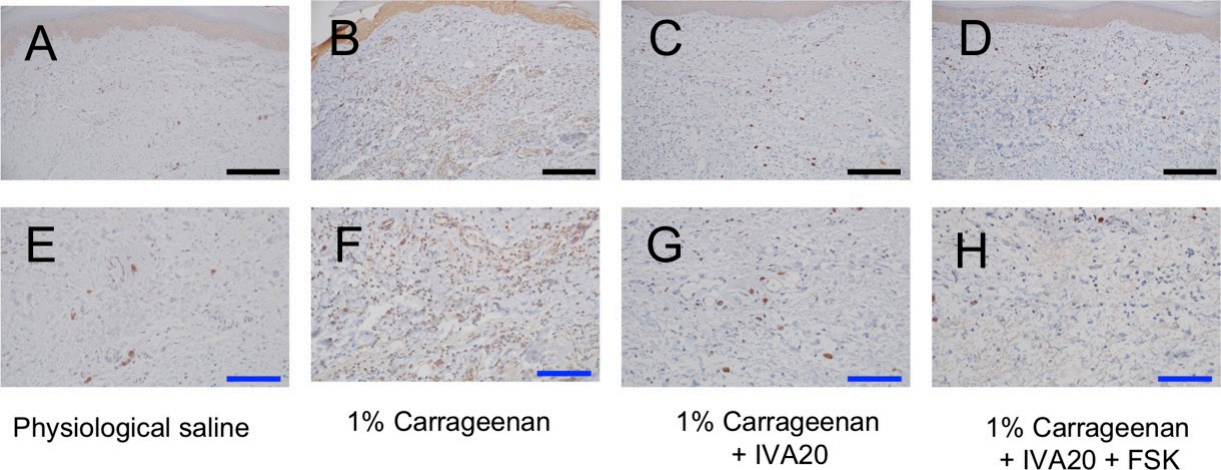
Immunohistochemical expression of TNF-alpha in the hindpaw area of rats (2 hours after injection). The following test solutions were subcutaneously injected into the hindpaw: physiological saline (A, E), 1% carrageenan (B, F), 1% carrageenan + ivabradine at 20 μM (IVA20) (C, G), and 1% carrageenan + IVA20 + forskolin at 10 μM (FSK) (D, H). A-D are shown at low magnification, and black scale bars represent 200 μm. E-H are shown at a higher magnification, and blue scale bars represent 100 μm.

In the samples collected 2 hours after the injection of carrageenan, greater expression of TNF-alpha based on immunohistochemistry was observed after the administration of physiological saline, while ivabradine at 20 and 50 μM reduced it. However, forskolin did not reverse the reduction of TNF-alpha expression caused by ivabradine (Fig 7).

### The effects of ivabradine and ZD7288 on LPS-stimulated TNF-alpha production in mouse macrophage-like cells

**Fig 8:**
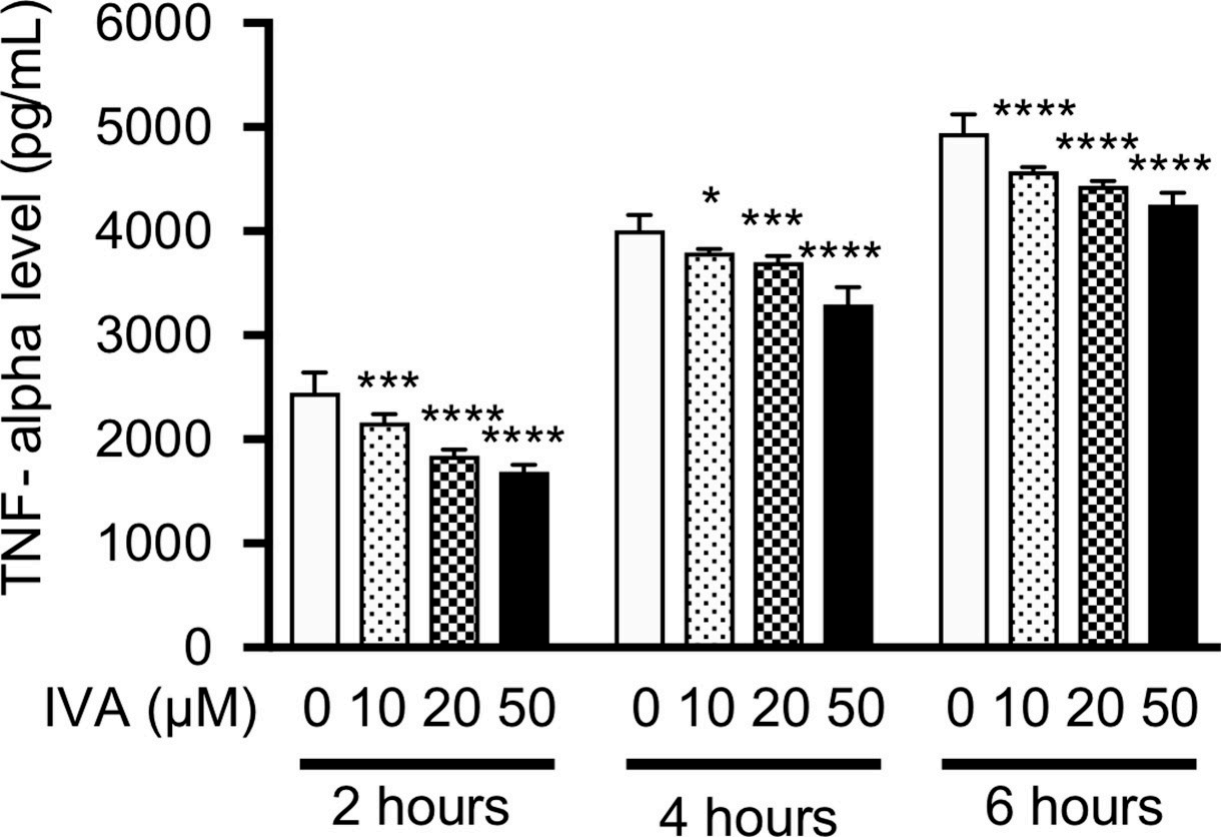
The effect of ivabradine (IVA) on TNF-alpha production in mouse macrophage-like cells. The cells were incubated with LPS at 10 ng/mL. TNF-alpha levels in the supernatants of the cells following 2-, 4-, and 6-hour incubation with only LPS, LPS + IVA at 10, 20, and 50 μM were measured. *P< 0.05, ***P< 0.001, ****P< 0.0001 compared with only LPS. Data represent the mean ± SD (n = 5 for each).

**Fig 9:**
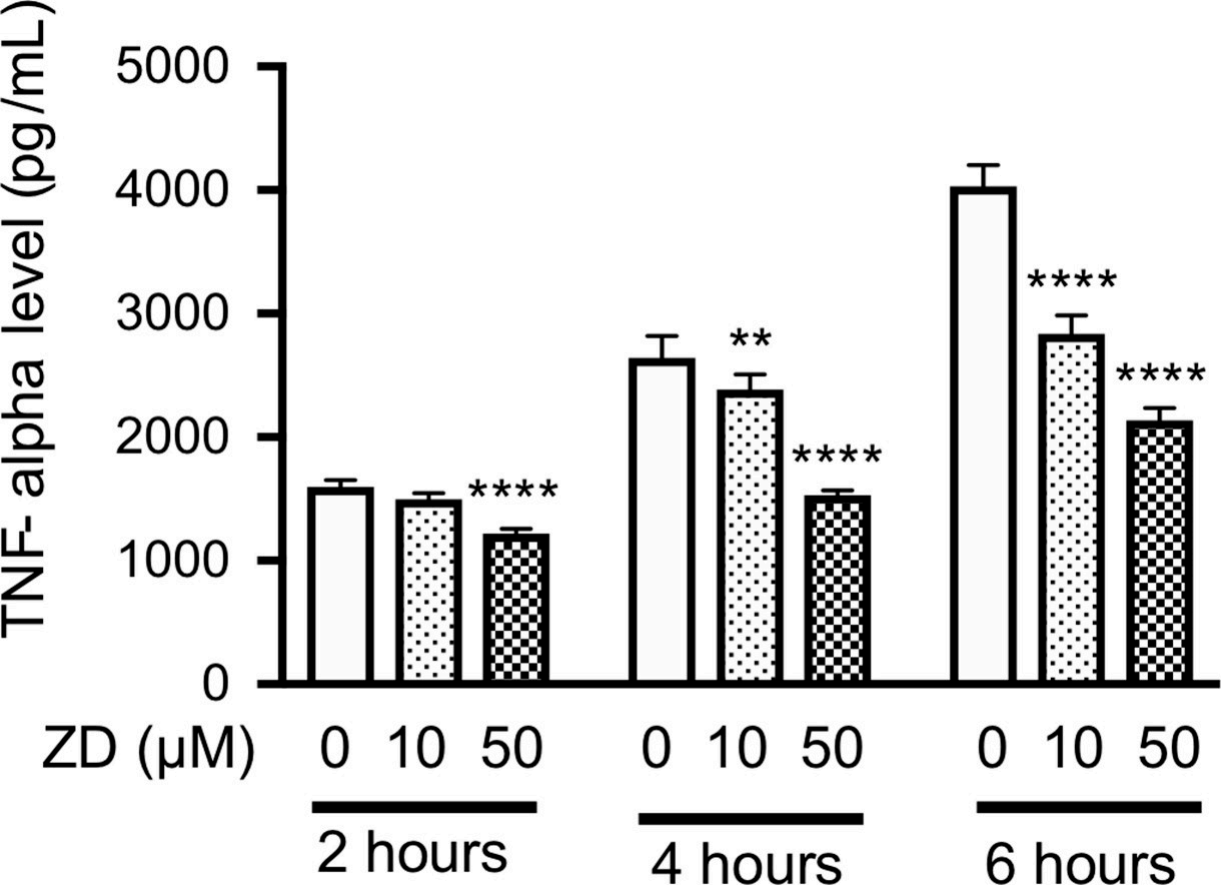
The effect of ZD7288 (ZD) on TNF production in mouse macrophage-like cells. The cells were incubated with LPS at 10 ng/mL. TNF-alpha levels in the supernatants of the cells following 2-, 4-, and 6-hour incubation with only LPS and LPS + ZD at 10 and 50 μM were measured. **P< 0.01, ****P< 0.0001 compared with only LPS. Data represent the mean ± SD (n = 5 for each).

**Fig 10:**
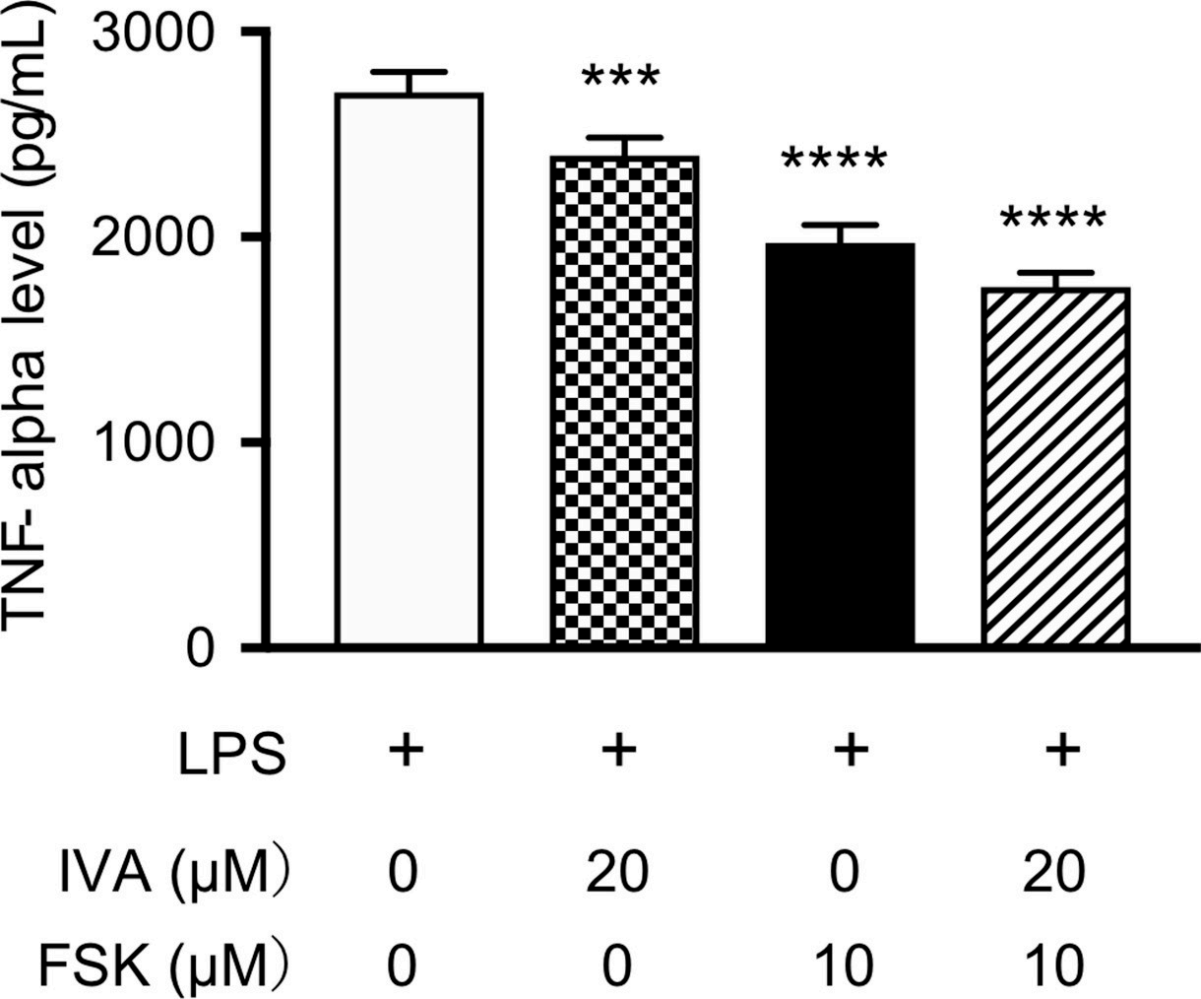
The effect of forskolin (FSK) to reverse the action of ivabradine (IVA) on LPS-stimulated TNF production in mouse macrophage-like cells. The cells were incubated with LPS at 10 ng/mL. TNF-alpha levels in the supernatants of the cells following 2-hour incubation with only LPS, LPS + IVA at 20 μM, LPS + FSK at 10 μM, and LPS + IVA at 20 μM + FSK at 10 μM were measured. ***P< 0.001, ****P< 0.0001 compared with only LPS. Data represent the mean ± SD (n = 5 for each).

In the incubated macrophage-like cells (RAW264.7 cells), ivabradine at 10, 20, and 50 μM dose-dependently inhibited LPS-stimulated TNF-alpha production on 4- and 6-hour incubation in the cells (Fig 8). ZD7288 at 10 and/or 50 μM also inhibited LPS-stimulated TNF-alpha production on 2-, 4-, and 6-hour incubation in the cells (Fig 9). However, there were no differences in TNF-alpha production among LPS plus ivabradine, LPS plus forskolin, and LPS plus ivabradine plus forskolin. Thus, forskolin at 10 μM did not reverse the inhibitory effect of ivabradine at 20 μM on TNF-alpha production in the cells (Fig 10).

## Discussion

The present study demonstrated that locally injected ivabradine is effective against carrageenan-induced inflammatory pain. This indicates that ivabradine may be a candidate for a new medicine to relieve inflammatory and neuropathic pain. In the present study, the local injection of ivabradine at a concentration of more than 20 μM significantly increased the pain threshold at the carrageenan-injected site of the hindpaw, and this effect was reversed by lamotrigine and forskolin. Lamotrigine directly activates HCN channels without elevating cAMP [32]. Forskolin is an adenylate cyclase activator that increases intracellular cAMP, which binds to a domain in the C-terminal tail on HCN channels [33], and increased cAMP results in the activation of HCN channels. Therefore, the results suggest that ivabradine acts on peripheral nerves and tissues via HCN channels. Forskolin was reported to have a greater effect on HCN activation than lamotrigine [33]. Forskolin also reversed the action of ivabradine as well as lamotrigine in the present study.

HCN1-4 channels are expressed in the hindpaws and peripheral sensory terminals of rats [22]. HCN1 and HCN2 channels are widely distributed in neurons, where they play roles in synaptic integration and neuronal excitability [34]. However, it was suggested that HCN1 channels do not play a marked role in either mechanical or heat-induced hyperalgesia following inflammation caused by the intraplantar injection of PGE2 [34]. Furthermore, the effects of the selective deletion of HCN2 channels on neuropathic pain in sensory neurons were much more marked, showing no enhanced sensitivity to heat, cold, or mechanical stimuli following nerve injury [24]. Therefore, these findings suggest that ivabradine acted via HCN2 channels in the inflammatory area of the rat hindpaw in the present study. This is consistent with the findings of other studies, suggesting that HCN2 channels play a role in neuropathic pain and that they are a target isoform [24, 35, 36].

The present study demonstrated that ivabradine had an inhibitory effect on not only neuropathic pain but also inflammatory responses in the hindpaw of rats, including the accumulation of leukocytes and TNF-alpha expression. Furthermore, ivabradine and ZD7288 had an inhibitory effect on the LPS-stimulated production of TNF-alpha in macrophage-like mouse cells (RAW264.7 cells) *in vitro*. HCN channels have been reported to be expressed in RAW264.7 cells [37]. The findings suggest that ivabradine has anti-inflammatory effects via HCN channels of inflammatory cells. Inflammatory mediators, which are released from inflammatory cells, are known to act on peripheral nerves and cause neuropathic pain. Therefore, it is thought that ivabradine can act not only on peripheral nerves via HCN2 channels but also on inflammatory cells around these peripheral nerves, resulting in the additive inhibition of inflammatory pain. If so, ivabradine’s effect may be important in the initial stage of inflammation.

On the other hand, cAMP is known to accelerate the activation of HCN1, HCN2, and HCN4 channels, but HCN3 channels are cAMP-independent [33]. Because forskolin increases intracellular cAMP, the finding that the effect of ivabradine was not reversed by forskolin in the incubated cells suggests that the anti-inflammatory effect would be neither via HCN1, HCN2, nor HCN4, but via HCN3 channels. However, because the activation rate of HCN1 channels by cAMP is less than that of HCN2 and HCN4 channels [33], we cannot fully exclude the possibility of the involvement of HCN1 channels. The direct roles of HCN1 and HCN3 channels in inflammation have not been clarified. Therefore, a new role of HCN1 or HCN3 channels in the peripheral tissues may have been identified in the present study.

It is important for clinical use for the effective dose to remain under the blood concentration that causes side effects. Ivabradine is clinically used as an anti-anginal and has been generally administered via intraoral or intraperitoneal routes in animal models. Although HCN channels in the CNS also play a crucial role in neuropathic pain [12, 20, 24], ivabradine does not cross the blood-brain barrier (BBB) [38]; therefore, it has no effect on the CNS. However, we need to consider that high-dose ivabradine administration could have side effects on peripheral organs, especially the heart. In past studies, ivabradine was intraperitoneally administered at a dose of more than 5 mg/kg [11]. The clinical dose (starting dose) of ivabradine as an anti-anginal is 2.5 mg twice daily [39]. The dose of 2.5 mg is equal to 0.05 mg/kg for a human weighing 50 kg. Therefore, the effective dose for neuropathic pain in past studies was much higher than the clinical dose. However, in the present study, we administered ivabradine at 20 μM to rats weighing 0.5 kg at a volume of 0.05 mL, resulting in a dose of 2 x 10^−6^ mg/kg, which is markedly lower than that used in other studies and the clinical dose. The clinical use of ivabradine is considered significant for relieving inflammatory and neuropathic pain.

The present study has the following limitations. The first is that we did not identify the isoform of HCN channels that mediates the effect of ivabradine in either *in vivo* or *in vitro* studies. To clearly demonstrate this, knock-out mice and knock-down cells would be necessary in *in vivo* and *in vitro* studies, respectively, because there are no products available that can specifically block each isoform of HCN channels. However, the purpose of the present study was to evaluate the effect of locally injected ivabradine on inflammatory pain, and we were able to demonstrate this. Further investigation on the identification of the isoform of HCN channels would be the next step. The finding that HCN1 or HCN3 channels may be involved in the anti-inflammatory action of HCN channel blockers may be worth further investigation.

The second is that we investigated mouse cells in an *in vitro* study while we used a rat model for an *in vivo* study. HCN channels have been demonstrated to distribute in the peripheral nerves in both animals [40]. However, there were no inflammatory-related cells of rats where HCN channel expression was demonstrated, while HCN channel expression was demonstrated in macrophage-like cells of the mouse (RAW264.7 cells). There were difference in the species between the studies; however, the difference is not considered to have significantly influenced the obtained findings.

The third is the possibility that the effect of ivabradine is generally induced at sites other than the injected site. This cannot be completely ruled out based on the present study. HCN channels are widely distributed throughout the whole body, and HCN1 channels are the most abundant in the dorsal root ganglia [20]. It is possible that injected ivabradine had an effect on sites other than the injected site in the periphery, but the concentration would be too low to significantly act on a target, as described above. It is reasonable to consider that ivabradine mainly acted on the injected site in the present study.

Forth is about using lamotrigine as an HCN activator. Lamotrigine inhibits sodium channels and is clinically used as an antiepileptic analgesic. So, using lamotrigine in the present experiments might make the results complex. However, we also used forskolin as another HCN activator, and the results obtained with the use of forskolin were the same as those with lamotrigine. Thus, the results indicate that the action of ivabradine is via the activation of HCN channels.

## Conclusion

The results of the present study demonstrated that locally injected ivabradine is effective against carrageenan-induced inflammatory pain via HCN channels. Its effect was considered to involve not only an action on peripheral nerves but also an anti-inflammatory effect. The findings suggest that the local injection of ivabradine may be useful for preventing the development of inflammatory and neuropathic pain.

## References

[1] Jensen MP, Chodroff MJ, Dworkin RH. The impact of neuropathic pain on health-related quality of life: review and implications. Neurology. 2007; 68(15): 1178–1182. 10.1212/01.wnl.0000259085.61898.9e 17420400

[2] Alleman CJ, Westerhout KY, Hensen M, Chambers C, Stoker M, Long S, et al Humanistic and economic burden of painful diabetic peripheral neuropathy in Europe: A review of the literature. Diabetes Res Clin Pract. 2015; 109(2): 215–225. 10.1016/j.diabres.2015.04.031 26008721

[3] Doth AH, Hansson PT, Jensen MP, Taylor RS. The burden of neuropathic pain: a systematic review and meta-analysis of health utilities. Pain. 2010; 149(2): 338–344. 10.1016/j.pain.2010.02.034 20227832

[4] Vinik A, Emir B, Cheung R, Whalen E. Relationship between pain relief and improvements in patient function/quality of life in patients with painful diabetic peripheral neuropathy or postherpetic neuralgia treated with pregabalin. Clin Ther. 2013; 35(5): 612–623. 10.1016/j.clinthera.2013.03.008 23541708

[5] Woolf CJ, Mannion RJ. Neuropathic pain: aetiology, symptoms, mechanisms, and management. Lancet. 1999; 353(9168): 1959–1964. 10.1016/S0140-6736(99)01307-0 10371588

[6] Schomberg D, Ahmed M, Miranpuri G, Olson J, Resnick DK. Neuropathic pain: role of inflammation, immune response, and ion channel activity in central injury mechanisms. Ann Neurosci. 2012; 19(3): 125–132. 10.5214/ans.0972.7531.190309 25205985 PMC4117080

[7] Sommer C, Leinders M, Üçeyler N. Inflammation in the pathophysiology of neuropathic pain. Pain. 2018; 159(3): 595–602. 10.1097/j.pain.0000000000001122 29447138

[8] Thacker MA, Clark AK, Marchand F, McMahon SB. Pathophysiology of peripheral neuropathic pain: immune cells and molecules. Anesth Analg. 2007; 105(3): 838–847. 10.1213/01.ane.0000275190.42912.37 17717248

[9] Baron R. Neuropathic pain: a clinical perspective. Handb Exp Pharmacol. 2009; 194: 3–30. 10.1007/978-3-540-79090-7_1 19655103

[10] Finnerup NB, Attal N, Haroutounian S, McNicol E, Baron R, Dworkin RH, et al Pharmacotherapy for neuropathic pain in adults: a systematic review and meta-analysis. Lancet Neurol. 2015; 14(2): 162–173. 10.1016/S1474-4422(14)70251-0 25575710 PMC4493167

[11] Young GT, Emery EC, Mooney ER, Tsantoulas C, McNaughton PA. Inflammatory and neuropathic pain are rapidly suppressed by peripheral block of hyperpolarisation-activated cyclic nucleotide-gated ion channels. Pain. 2014; 155(9): 1708–1719. 10.1016/j.pain.2014.05.021 24861581

[12] Takasu K, Ono H, Tanabe M. Spinal hyperpolarization-activated cyclic nucleotide-gated cation channels at primary afferent terminals contribute to chronic pain. Pain. 2010; 151(1): 87–96. 10.1016/j.pain.2010.06.020 20619969

[13] Santoro B, Grant SG, Bartsch D, Kandel ER. Interactive cloning with the SH3 domain of N-src identifies a new brain specific ion channel protein, with homology to Eag and cyclic nucleotide-gated channels. Proc Natl Acad Sci USA. 1997; 94(26): 14815–14820. 10.1073/pnas.94.26.14815 9405696 PMC25120

[14] Santoro B, Liu DT, Yao H, Bartsch D, Kandel ER, Siegelbaum SA, et al Identification of a gene encoding a hyperpolarization-activated pacemaker channel of brain. Cell. 1998; 93(5): 717–729. 9630217 10.1016/s0092-8674(00)81434-8

[15] Santoro B, Tibbs GR. The HCN gene family: molecular basis of the hyperpolarization-activated pacemaker channels. Ann N Y Acad Sci. 1999; 868: 741–764. 10414361 10.1111/j.1749-6632.1999.tb11353.x

[16] Ludwig A, Zong X, Jeglitsch M, Hofmann F, Biel M. A family of hyperpolarization-activated mammalian cation channels. Nature. 1998; 393(6685): 587–591. 10.1038/31255 9634236

[17] Pape HC. Queer current and pacemaker: the hyperpolarization-activated cation current in neurons. Annu Rev Physiol. 1996; 58: 299–327. 10.1146/annurev.ph.58.030196.001503 8815797

[18] Robinson RB, Siegelbaum SA. Hyperpolarization-activated cation currents: from molecules to physiological function. Annu Rev Physiol. 2003; 65: 453–480. 10.1146/annurev.physiol.65.092101.142734 12471170

[19] Chaplan SR, Guo HQ, Lee DH, Luo L, Liu C, Kuei C, et al Neuronal hyperpolarization-activated pacemaker channels drive neuropathic pain. J Neurosci. 2003; 23(4): 1169–1178. 12598605 10.1523/JNEUROSCI.23-04-01169.2003 PMC6742242

[20] Sartiani L, Mannaioni G, Masi A, Romanelli MN, Cerbai E. The hyperpolarization-activated cyclic nucleotide-gated channels: from biophysics to pharmacology of a unique family of ion channels. Pharmacol Rev. 2017; 69(4): 354–395. 10.1124/pr.117.014035 28878030

[21] Postea O, Biel M. Exploring HCN channels as novel drug targets. Nat Rev Drug Discov. 2011; 10(12): 903–914. 10.1038/nrd3576 22094868

[22] Luo L, Chang L, Brown SM, Ao H, Lee DH, Higuera ES, et al Role of peripheral hyperpolarization-activated cyclic nucleotide-modulated channel pacemaker channels in acute and chronic pain models in the rat. Neuroscience. 2007; 144(4): 1477–1485. 10.1016/j.neuroscience.2006.10.048 17196750

[23] DiFrancesco D, Camm JA. Heart rate lowering by specific and selective I(f) current inhibition with ivabradine: a new therapeutic perspective in cardiovascular disease. Drugs. 2004; 64(16): 1757–1765. 10.2165/00003495-200464160-00003 15301560

[24] Emery EC, Young GT, Berrocoso EM, Chen L, McNaughton PA. HCN2 ion channels play a central role in inflammatory and neuropathic pain. Science. 2011; 333(6048): 1462–1466. 10.1126/science.1206243 21903816

[25] Schnorr S, Eberhardt M, Kistner K, Rajab H, Käßer J, Hess A, et al HCN2 channels account for mechanical (but not heat) hyperalgesia during long-standing inflammation. Pain. 2014; 155(6): 1079–1090. 10.1016/j.pain.2014.02.006 24525276

[26] Jagels MA, Hugli TE. Mechanisms and mediators of neutrophilic leukocytosis. Immunopharmacology. 1994; 28(1): 1–18. 7928299 10.1016/0162-3109(94)90034-5

[27] Di Rosa M, Giroud JP, Willoughby DA. Studies on the mediators of the acute inflammatory response induced in rats in different sites by carrageenan and turpentine. J Pathol. 1971; 104(1): 15–29. 10.1002/path.1711040103 4398139

[28] Honda Y, Higuchi H, Matsuoka Y, Yabuki-Kawase A, Ishii-Maruhama M, Tomoyasu Y, et al The inhibitory effect of locally injected dexmedetomidine on carrageenan-induced nociception in rats. Eur J Pharmacol. 2015; 764: 215–219. 10.1016/j.ejphar.2015.06.054 26160316

[29] Poolos NP, Migliore M, Johnston D. Pharmacological upregulation of h-channels reduces the excitability of pyramidal neuron dendrites. Nat Neurosci. 2002; 5(8): 767–774. 10.1038/nn891 12118259

[30] Chaplan SR, Bach FW, Pogrel JW, Chung JM, Yaksh TL. Quantitative assessment of tactile allodynia in the rat paw. J Neurosci Methods. 1994; 53(1): 55–63. 7990513 10.1016/0165-0270(94)90144-9

[31] Sukegawa S, Higuchi H, Inoue M, Nagatsuka H, Maeda S, Miyawaki T. Locally injected dexmedetomidine inhibits carrageenin-induced inflammatory responses in the injected region. Anesth Analg. 2014; 118(2): 473–480. 10.1213/ANE.0000000000000060 24445644

[32] Zhong N, Zucker RS. cAMP acts on exchange protein activated by cAMP/cAMP-regulated guanine nucleotide exchange protein to regulate transmitter release at the crayfish neuromuscular junction. J Neurosci. 2005; 25(1): 208–214. 10.1523/JNEUROSCI.3703-04.2005 15634783 PMC6725206

[33] Wainger BJ, DeGennaro M, Santoro B, Siegelbaum SA, Tibbs GR. Molecular mechanism of cAMP modulation of HCN pacemaker channels. Nature. 2001; 411(6839): 805–810. 10.1038/35081088 11459060

[34] Momin A, Cadiou H, Mason A, McNaughton PA. Role of the hyperpolarization-activated current Ih in somatosensory neurons. J Physiol. 2008; 586(24): 5911–5929. 10.1113/jphysiol.2008.163154 18936078 PMC2655434

[35] Emery EC, Young GT, McNaughton PA. HCN2 ion channels: an emerging role as the pacemakers of pain. Trends Pharmacol Sci. 2012; 33(8): 456–463. 10.1016/j.tips.2012.04.004 22613784

[36] Tsantoulas C, Laínez S, Wong S, Mehta I, Vilar B, McNaughton PA. Hyperpolarization-activated cyclic nucleotide-gated 2 (HCN2) ion channels drive pain in mouse models of diabetic neuropathy. Sci Transl Med. 2017; 9(409): eaam6072. 10.1126/scitranslmed.aam6072 PMC5720342 28954930

[37] Notomi T, Kuno M, Hiyama A, Ohura K, Noda M, Skerry TM. Zinc-induced effects on osteoclastogenesis involves activation of hyperpolarization-activated cyclic nucleotide modulated channels via changes in membrane potential. J Bone Miner Res. 2015; 30(9): 1618–1626. 10.1002/jbmr.2507 25762086

[38] Savelieva I, Camm AJ. Novel If current inhibitor ivabradine: safety considerations. Adv Cardiol. 2006; 43: 79–96. 10.1159/000095430 16936474

[39] Prescribing information. CORLANOR®(ivabradine) tablets. revised: 2017 Jan [cited 10 Oct 2018]. available from: https://pi.amgen.com/~/media/amgen/repositorysites/pi-amgen-com/corlanor/corlanor_pi.pdf

[40] Romanelli MN, Sartiani L, Masi A, Mannaioni G, Manetti D, Mugelli A, et al HCN channels modulators: the need for selectivity. Curr Top Med Chem. 2016; 16(16): 1764–1791. 10.2174/1568026616999160315130832 26975509 PMC5374843

